# Diagnosing and Treating Periodontitis and Multimorbidity Altogether: The Case Example of the Physical Exercise Hormone Irisin

**DOI:** 10.1111/jre.70081

**Published:** 2026-03-08

**Authors:** Larysa Saboya Cavalotti Perdonsini, Rafael Scaf de Molon, Søren Jepsen, Iain Chapple, Joao Paulo Steffens

**Affiliations:** ^1^ Postgraduate Program in Dentistry Universidade Federal Do Paraná Curitiba Paraná Brazil; ^2^ School of Dentistry at Araçatuba Universidade Estadual Paulista Araçatuba São Paulo Brazil; ^3^ Department of Periodontology, Operative and Preventive Dentistry University of Bonn Bonn Germany; ^4^ School of Health Sciences, College of Medicine and Health University of Birmingham Birmingham UK; ^5^ Birmingham NIHR Biomedical Research Centre in Inflammation University of Birmingham Birmingham UK

**Keywords:** cytokines, myokines, non‐communicable diseases, periodontitis

## Abstract

Irisin is an extracellular peptide stimulated by exercise and could act as both a biomarker and mediate the positive impact of exercise. This scoping review synthesizes human evidence on irisin variations in non‐communicable diseases, showing an increase of irisin in saliva and gingival tissues in periodontitis, potentially reflecting reparative or immunomodulatory activity.

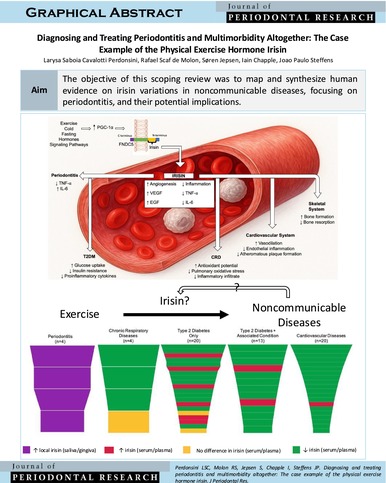

## 1 Introduction

Irisin is an extracellular peptide cleaved from the transmembrane protein fibronectin type III domain‐containing protein 5 (FNDC5) through a process regulated by peroxisome‐proliferator‐activated receptor‐gamma coactivator 1‐alpha (PGC‐1α), a key transcriptional factor also stimulated by exercise [1]. First described in 2012, irisin is predominantly expressed in skeletal muscle and adipose tissue, with additional expression in bone, brain, periodontal tissues, and dental pulp [1, 2]. Given this dual metabolic and immunomodulatory profile, irisin has gained attention as a potential mediator connecting exercise, chronic inflammation, and periodontal health (Supporting Information SM1) [2, 3].

Mechanistically, irisin promotes the browning of white adipose tissue, enhances thermogenesis, and increases energy expenditure [1]. Beyond metabolism, exogenous irisin administration, similarly to physical exercise, positively impacted bone metabolism [3]. By contrast, increased endogenous levels of gingival irisin have been reported during progression of ligature‐induced periodontitis in rats—a phenomenon termed ‘the irisin paradox’, [2] whose mechanism remains largely unknown.

Exercise has been proposed as a beneficial adjunctive strategy in non‐communicable diseases (NCDs), including periodontitis [4]. Therefore, beyond serving as a biomarker, irisin could also mediate the positive impact of exercise on inflammation. The objective of this scoping review was to map and synthesize human evidence on irisin variations in NCDs, focusing on periodontitis, and their potential implications.

## 2 Methods

This review is based on a pre‐registered protocol that also included other NCDs (SM2). For the exposure ‘periodontitis’, the original PubMed search was expanded to Embase, Scopus and Web of Science, and updated in November 2025. Risk of bias was assessed using the checklist for analytical cross‐sectional studies (JBI, https://jbi.global/critical‐appraisal‐tools).

## 3 Results and Discussion

Four clinical studies met the inclusion criteria for periodontitis, all with cross‐sectional designs (SM3) [5, 6, 7, 8]. Overall, studies consistently reported increased local irisin levels in saliva (*n* = 3) or gingival tissue (*n* = 1) in individuals with periodontitis, while one study reported no significant differences in serum [6]. The studies assessing saliva used similar collection protocols, including fasting and avoidance of recent oral hygiene procedures. Salivary irisin correlated positively with periodontal clinical parameters and with interleukin‐6 [6, 7]. Irisin elevation appeared greater in stage III periodontitis [6, 7], suggesting an association between disease severity and local irisin upregulation. A summary of the findings of each included study is shown in Table 1. Risk of bias assessment indicated major concerns related to confounding factors (Table S2), as all studies lacked the identification and stratification according to confounders, in particular exercise. Multivariable regressions were not reported.

**TABLE 1:** Summary of the included studies analyzing irisin levels in individuals with periodontitis.

Author/year	Population/study location	Intervention/exposure	Control	Methods	Main findings	Conclusions	Study design
Khan et al. (2022)	40 patients from a university setting in Pakistan	20 patients with generalized chronic periodontitis (4 females and 16 males): PD ≥ 5 mm and CAL ≥ 2 mm Mean age: 43.3 ± 6.1	20 periodontally healthy patients (10 females and 10 males) Mean age: 37.6 ± 2.6	– Unstimulated saliva – 8–10 am – Rinsed distilled water for 1 min – Passive drooling – Frozen at −80°C – ELISA: Human FNDC5 Elabscience, E‐EL‐H2254	Saliva Controls: 4.0 ± 2.5 ng/mL Periodontitis: 6.8 ± 4.0 ng/mL *p*‐value: 0.009	Positive but non‐significant correlation between salivary irisin levels and clinical parameters in each individually analyzed group, except for plaque percentage in healthy controls	Cross‐sectional
Turkmen et al. (2023)	40 patients from a university setting in Turkey	20 patients with generalized stage III grade B periodontitis (9 females and 11 males) Mean age: 41.1 ± 6.4	20 periodontally healthy patients (11 females and 9 males) Mean age: 34.2 ± 7.4	– Serum and unstimulated saliva – 8–10 am – Saliva centrifuged at 10000 rpm (10 min) – Blood centrifuged at 4000 rpm (10 min) – Frozen at −80°C – ELISA: Elabscience	Serum Controls: 929.4 ± 371.1 pg/mL Periodontitis: 1033.0 ± 466.9 pg/mL *p*‐value: 0.49 Saliva Controls: 8.3 ± 12.7 pg/mL Periodontitis: 27.6 ± 41.6 pg/mL *p*‐value: < 0.001	Significant positive correlation between salivary irisin, IL‐6, and clinical parameters. Positive correlation between BMI and IL‐6 levels	Cross‐sectional
Saribas et al. (2025)	66 patients from a university setting in Turkey	33 patients with stage III grade C periodontitis (17 females and 16 males) Mean age: 45.7 ± 6.8	33 periodontally healthy patients (18 females and 15 males) Mean age: 28.7 ± 5.2	– Unstimulated whole saliva – 10 am–12 pm – Passive drooling – Frozen at −80°C – ELISA: Human Irisin – SunRed, 201‐12‐5328	Saliva Controls: 9.9 ± 3.1 ng/mL Periodontitis: 28.9 ± 8.0 ng/mL *p*‐value: < 0.001	Salivary irisin and IL‐6 significantly elevated in periodontitis patients, aligning with clinical parameters. In silico analyses revealed potential interactions between IL‐6 and irisin	Cross‐sectional
Yang et al. (2025)	20 patients from a university setting in China	10 patients with any stage of periodontitis, according to current classification	10 periodontally healthy patients	– Gingival samples – Relative expression of FNDC5 mRNA – IHC and IF staining	– Relative expression of FNDC5 mRNA ≈12× higher in periodontitis patients – Immunostaining ≈4× higher in periodontitis patients	Spearman correlation analysis revealed a correlation between irisin mRNA levels and clinical periodontal parameters, especially with PD	Cross‐sectional

Abbreviations: BMI, body mass index; CAL, clinical attachment loss; IF, immunofluorescence; IHC, immunohistochemistry; PD, probing depth.

Additionally, the measurement of irisin was heterogeneous regarding analytical technique (ELISA, RT‐PCR, and immunohistochemistry) and protein specification (FNDC5 or irisin). Importantly, consistent findings were observed regardless of the technique, suggesting that the once reported cross‐reactivity [9] could be overcome by the multiplicity of tests used. Depending on the protein specification, salivary levels of irisin in controls varied up to 1000‐fold. Analytical standardization remains an issue in the field.

Increased local irisin levels likely reflect a pro‐resolution role, as suggested by its association with growth factors during periodontal repair [2]. Hence, beyond its potential role as a biomarker, irisin may possibly serve as a relevant mediator to tackle periodontitis‐associated multimorbidity. For example, in addition to the benefits of exercise for overweight/obesity, a recent meta‐analysis reported that training improved circulating irisin levels [10], which positively impacts bone health [3]. In addition, preclinical evidence suggests irisin administration has anti‐inflammatory and regenerative effects on periodontal tissues, including reduction of pro‐inflammatory cytokines such as TNF‐α [2]. Therefore, integrating structured exercise into periodontal care could possibly benefit systemic disease risk, aligning with current recommendations for holistic management of chronic conditions [4]. However, the impact of exercise on irisin levels in individuals with periodontitis remains unknown.

These localized increases contrast with the overall systemic reduction in circulating irisin frequently reported in type‐2 diabetes mellitus, cardiovascular, and chronic respiratory diseases (SM4‐8). In those contexts, low irisin levels correlate with insulin resistance, dyslipidaemia, endothelial dysfunction, reduced physical performance, and adverse cardiometabolic outcomes. This divergence supports the concept of compartment‐specific regulation shaped by the local inflammatory and metabolic microenvironment.

Future investigations should prioritize stratification by confounders such as age, sex, health status, common risk factors, and physical exercise. Besides, mechanisms relating irisin, growth factors, TNF‐α, and oxidative stress should be explored [2, 4]. Detailed comparisons across saliva, gingival tissue, and serum or plasma collected under strict protocols and analyzed using validated standardised assays are essential to clarify the biological meaning of irisin fluctuations. Longitudinal and interventional studies will be critical to determine causality and whether irisin can serve as a predictive biomarker of disease progression or a therapeutic target in periodontal and systemic health contexts.

## 4 Conclusion

This review highlights the complex and context‐dependent behavior of irisin. In periodontitis, limited evidence suggests that irisin demonstrates a localized increase in saliva and gingival tissues, possibly reflecting reparative or immunomodulatory activity. Clinically, salivary irisin could emerge as both a non‐invasive biomarker for periodontal inflammation and an integrative indicator linking periodontitis with broader metabolic health.

## Funding

This study received no specific funding. RSM is currently supported by a grant provided by Sao Paulo Research Foundation (FAPESP), #2023/15750‐7.

## Supporting information


**Data S1:** jre70081‐sup‐0001‐Supinfo1.docx.

**Table S2:** Risk of bias assessment of the included studies analysing irisin levels in individuals with periodontitis.
