# Supplementary material for: Diagnosing and Treating Periodontitis and Multimorbidity Altogether: The Case Example of the Physical Exercise Hormone Irisin
Source: J Periodontal Res. 2026 Mar 8;61(6):733–5. doi: 10.1111/jre.70081 (PMC13472005; doi:10.1111/jre.70081)
Supplement: Supplementary file 1 [file JRE-61-733-s001.docx]

**Table S2.** Risk of bias assessment of the included studies analysing irisin levels in individuals with periodontitis.

| Author, year | Inclusion criteria | Participants and settings | Measurement of exposure | Measurement of condition | Identification of confounding factors | Strategies for confounding factors | Measurement of outcome | Statistical analysis |
| --- | --- | --- | --- | --- | --- | --- | --- | --- |
| Khan et al., 2022 | Yes | Yes | N/A | Yes | Unclear | No | Yes | Unclear |
| Turkmen et al., 2023 | Yes | Unclear | N/A | Yes | Yes | No | Yes | Yes |
| Saribas et al., 2025 | Yes | Yes | N/A | Yes | Yes | No | Yes | Yes |
| Yang et al., 2025 | Yes | Unclear | N/A | Yes | Unclear | No | Yes | Yes |

N/A: Not applicable. Condition: Periodontitis. Outcome: Irisin.
