# Supplementary material for: Diagnosing and Treating Periodontitis and Multimorbidity Altogether: The Case Example of the Physical Exercise Hormone Irisin
Source: J Periodontal Res. 2026 Mar 8;61(6):733–5. doi: 10.1111/jre.70081 (PMC13472005; doi:10.1111/jre.70081)
Supplement: Supplementary file 2 [file JRE-61-733-s002.docx]

**Diagnosing and Treating Periodontitis and Multimorbidity Altogether: The Case Example of the Physical Exercise Hormone Irisin**

Larysa Saboya Cavalotti Perdonsini^1^, Rafael Scaf de Molon^2^, Søren Jepsen,^3^ Iain Chapple,^4,5^ Joao Paulo Steffens^1^

^1^ Postgraduate Program in Dentistry. Universidade Federal do Paraná, Curitiba, PR, Brazil

^2^ School of Dentistry at Araçatuba, Universidade Estadual Paulista, Araçatuba, PR, Brazil

^3^ Department of Periodontology, Operative and Preventive Dentistry. University of Bonn, Bonn, Germany

^4^ School of Health Sciences, College of Medicine and Health. University of Birmingham, Birmingham, UK

^5^ Birmingham NIHR Biomedical Research Centre in Inflammation, Birmingham, UK

**Corresponding Author**

Dr. Joao Paulo Steffens. 632 Pref. Lothário Meissner Av. Postal code: 80210-170. Curitiba – PR – Brazil. E-mail: joao.steffens@ufpr.br.

**SUPPLEMENTAL MATERIAL (SM)**

**Table of Contents**

SM1. Schematic representation of the impact of irisin in different systems and diseases…………………………………..……….……..…………..03

SM2. Methods: Scoping review on the association between circulating irisin levels and common noncommunicable diseases [cardiovascular diseases (CVDs), chronic respiratory diseases (CRDs) and type 2 diabetes mellitus (T2DM)]………………………………..…………………………04

SM3. Flowchart of the identification, screening, eligibility, inclusion and analysis process of studies in the scoping review for periodontitis…….….08

SM4. Flowchart of the identification, screening, eligibility, inclusion and analysis process of studies in the scoping review for type 2 diabetes mellitus (T2DM), cardiovascular diseases (CVD) and chronic respiratory diseases (CRD)………………………………………………………………09

SM5. Summary of the included studies analyzing irisin levels in individuals with chronic respiratory diseases………..…………….………………10

SM6. Summary of the included studies analyzing irisin levels in individuals with type 2 diabetes……………………………………………………..11

SM7. Summary of the included studies analyzing irisin levels in individuals with cardiovascular diseases…………………………………………..21

SM8. Funnel plots of included studies within each condition……………………………………………………………………………………………….29

References……………………………………………………………………………………………………………………………………………………….30

**SM1.** Schematic representation of the impact of irisin in different systems and diseases. After the stimulation of peroxisome-proliferator-activated receptor-gamma coactivator 1-alpha (PGC-1α), the fibronectin type III domain-containing protein 5 (FNDC5) is cleaved in its extracellular domain to release irisin. From circulation, irisin can also reach distant tissues and organs to exert its effects. VEGF: vascular endothelial growth factor. EGF: endothelial growth factor. IL-6: interleukin-6. T2DM: Type 2 diabetes mellitus. CRD: Chronic respiratory diseases.

**SM2.** **Methods: Scoping review on the association between circulating irisin levels and common noncommunicable diseases (NCDs) [periodontitis, cardiovascular diseases (CVDs), chronic respiratory diseases (CRDs) and type 2 diabetes mellitus (T2DM)]**

*Protocol and registration*

The methodological framework for this scoping review was based on Arksey and O’Malley with enhancements recommended by the Joanna Briggs Institute and the PRISMA extension for scoping reviews (PRISMA-ScR). This review also followed the procedures described in the EQUATOR network website and was registered in the Open Science Framework (OSF) database, doi: 10.17605/OSF.IO/2XZJP.

*Objectives and focused question*

The objective of this scoping review was to map and synthesize existing human clinical evidence on alterations of irisin levels in selected NCDs. The review process encompassed a systematic search, transparent study selection, structured data charting, and narrative synthesis to address the guiding question: “*Are irisin levels increased or decreased in NCDs?*”

*Information sources*

Information sources and the search strategy were developed to capture all relevant human studies without temporal restriction. The PubMed database was queried using combinations of the keyword “irisin” with Medical Subject Headings (MeSH) terms representing the conditions of interest: “Periodontitis,” “Type 2 Diabetes Mellitus,” “Cardiovascular Diseases,” and “Respiratory Tract Diseases”. Search filters limited retrieval to studies involving human participants and publications in English. The full detailed search strategy (SS) is provided below. All retrieved records were exported to EndNote reference management software, where duplicate entries were identified and removed prior to screening.

**SS1:** (("irisin"[All Fields] OR "irisin s"[All Fields]) AND ("diabetes mellitus, type 2"[MeSH Terms] OR "type 2 diabetes mellitus"[All Fields])) AND ((humans[Filter]) AND (english[Filter]))

**SS2:** (("irisin"[All Fields] OR "irisin s"[All Fields]) AND ("cardiovascular diseases"[MeSH Terms] OR ("cardiovascular"[All Fields] AND "diseases"[All Fields]) OR "cardiovascular diseases"[All Fields])) AND ((humans[Filter]) AND (english[Filter]))

**SS3:** (("irisin"[All Fields] OR "irisin s"[All Fields]) AND (("chronic"[All Fields] OR "chronical"[All Fields] OR "chronically"[All Fields] OR "chronicities"[All Fields] OR "chronicity"[All Fields] OR "chronicization"[All Fields] OR "chronics"[All Fields]) AND ("respiratory tract diseases"[MeSH Terms] OR ("respiratory"[All Fields] AND "tract"[All Fields] AND "diseases"[All Fields]) OR "respiratory tract diseases"[All Fields] OR ("respiratory"[All Fields] AND "diseases"[All Fields]) OR "respiratory diseases"[All Fields]))) AND ((humans[Filter]) AND (english[Filter]))

**SS4:** (("irisin"[All Fields] OR "irisin s"[All Fields]) AND ("periodontal"[All Fields] OR "periodontally"[All Fields] OR "periodontically"[All Fields] OR "periodontics"[MeSH Terms] OR "periodontics"[All Fields] OR "periodontic"[All Fields] OR "periodontitis"[MeSH Terms] OR "periodontitis"[All Fields] OR "periodontitides"[All Fields])) AND ((humans[Filter]) AND (english[Filter]))

The search for general NCDs was last updated in October 2025. The search for periodontitis was last updated in November 2025 and expanded to Embase, Scopus and Web of Science databases.

*Study Selection and Exclusion Criteria*

Eligibility criteria included original primary research studies involving human subjects that reported measurements of circulating or local irisin levels in populations diagnosed with periodontitis, CVDs, CRDs, or T2DM. Observational study designs (cross-sectional, case-control, cohorts) and interventional studies with appropriate baseline or comparator data were considered. Studies were required to include a comparator group without the condition – this could be either a healthy control group or a clearly defined reference without the investigated condition. Exclusion criteria encompassed studies solely reporting association data between irisin and other biomarkers, without reporting numerical values for their levels.

*Search strategy and selection process*

Study selection proceeded in two phases. Titles and abstracts of de-duplicated records were independently screened by two reviewers to identify potentially relevant articles (LSCP and JPS). Full texts of studies passing the initial screen were retrieved and assessed in detail against the eligibility criteria by the same pair of reviewers. Discrepancies in both screening and full-text eligibility decisions were resolved through consensus discussion, and if necessary, by consultation with a third reviewer (RSdM). Reasons for exclusion at the full-text stage were documented.

*Data extraction and collection process*

Data extraction was carried out using a standardized and piloted charting form implemented in Microsoft Excel and structured according to the Population, Exposure, Comparison, Outcome, and Study design (PECOS) strategy. The form was evaluated on a subset of included studies to ensure clarity and consistency between reviewers. Extracted information comprised bibliographic details (authors, year, geographic location), study design, characteristics of the study population (sample size, age distribution, sex, diagnostic criteria and definitions for the disease of interest, inclusion and exclusion criteria), description of the comparator or control group, type of biological sample analyzed (e.g., serum, plasma, saliva, gingival crevicular fluid), methods used for irisin quantification (assay type, manufacturer, sensitivity, sample handling and processing protocols, when available) and principal reported findings. Specific outcomes recorded included the direction and magnitude of irisin level alterations, statistical significance, correlations with disease severity indices, inflammatory markers, and other relevant clinical parameters.

*Synthesis methods*

Synthesis of results was descriptive. Studies were organized by disease category, and within each category, summaries highlighted patterns in irisin level alterations, distinctions between systemic versus local measurements, and methodological heterogeneity. Where available, secondary analyses such as associations of irisin with inflammatory biomarkers, metabolic parameters, and measures of disease severity were integrated.

**SM3.** Flowchart of the identification, screening, eligibility, inclusion and analysis process of studies in the scoping review for periodontitis.

**SM4.** Flowchart of the identification, screening, eligibility, inclusion and analysis process of studies in the scoping review for type 2 diabetes mellitus (T2DM), cardiovascular diseases (CVD) and chronic respiratory diseases (CRD).

**SM5. Summary of the included studies analyzing irisin levels in individuals with chronic respiratory diseases (CRDs).**

| Author/Year | Population/Study location | Intervention/ Exposure | Control | Outcome | Conclusions | Study design |  |
| --- | --- | --- | --- | --- | --- | --- | --- |
| Chronic Obstructive Pulmonary Disease (COPD) | | | | | | | |
| Ijiri et al., 2015 | 99 subjects from a university setting in Japan | 72 patients with COPD | 27 control subjects | ↓ Serum irisin in COPD patients compared to healthy controls. | Significant correlation between irisin levels and physical activity in all subjects, irisin levels increased after 8-week exercise training | Cross-sectional |  |
| Cuttitta et al., 2023 | 51 patients from Italy | 25 patients with COPD | 26 healthy controls | → Serum irisin levels in COPD patients and healthy controls. | In COPD patients, there was a significant inverse correlation between irisin, as well as a negative correlation between haptoglobin and irisin. | Cross-sectional |  |
| Other CRDs | | | | | | | |
| Shiotani et al., 2022 | 59 patients who underwent lung transplantation at a university setting in Japan | With chronic graft dysfunction after living donor lung lobe transplantation (LDLLT-CLAD; n=11) With chronic graft dysfunction after deceased donor lung transplantation (CLT-CLAD; n=7) | Without chronic lung graft dysfunction (non-CLAD; n=41) | ↓ Plasma irisin levels in LDLLT-CLAD patients compared to non-CLAD. | Significant association between emphysematous changes and CLAD severity in both LDLLT and CLT recipients | Retrospective cohort | |
| Kaya et al., 2024 | 176 individuals from a university setting in Turkey | 90 patients with sarcoidosis | 86 healthy individuals | ↓ Serum irisin in sarcoidosis patients compared to healthy controls. | Irisin was significantly lower in sarcoidosis. May serve as a potential biomarker. No significant difference in adiponectin levels. | Case-control | |

**SM6. Summary of the included studies analyzing irisin levels in individuals with type 2 diabetes.**

| Author/Year | Population/Study location | Intervention/ Exposure | Control | Outcome | Conclusions | Study design |
| --- | --- | --- | --- | --- | --- | --- |
| Type 2 Diabetes Only - Observational | | | | | | |
| Choi et al., 2013 | 208 participants from South Korea | 104 T2DM patients | 104 healthy controls with normal glucose tolerance | ↓ Serum irisin levels in T2DM patients compared to controls. | An inverse association was observed between irisin levels and newly diagnosed T2DM. Higher irisin levels were linked to reduced odds of newly diagnosed diabetes | Cross-sectional |
| Xiang et al., 2014 | 228 participants from China | 188 T2DM patients | 40 healthy controls | ↓ Serum irisin levels in T2DM patients compared to controls. | Positive correlation between irisin levels and flow-mediated dilation (FMD), suggesting a protective role of irisin in endothelial function | Cross-sectional |
| Alis et al., 2014 | 144 participants from a university setting in Spain | 69 T2DM patients | 75 euglycemic controls | ↓ Serum irisin levels in T2DM patients than in euglycemic controls. | Inverse correlation between irisin and homocysteine levels; as homocysteine levels increased, irisin levels decreased | Cross-sectional |
| Wang et al., 2015 | 200 participants from China | 100 T2DM patients | 100 healthy controls | ↓ Serum irisin levels in T2DM than in controls | Positive correlation between irisin levels and flow-mediated dilation (FMD); negative correlation with urinary albumin excretion (UAE), indicating potential endothelial protective effects | Cross-sectional |
| Duran et al., 2015 | 263 sedentary women from Turkey | 50 T2DM patients, 65 with both impaired fasting glucose and impaired glucose tolerance (IFG + IGT), 60 with isolated impaired fasting glucose (IFG), and 36 with isolated impaired glucose tolerance (IGT) | 52 normal glucose tolerance (NGT) controls | ↓ Serum irisin in IFG + IGT and T2DM than in NGT. | Negative correlations between irisin and BMI, LDL-C, triglycerides, and postprandial glucose; positive correlation with HDL-C. Irisin levels decreased progressively with worsening glucose tolerance | Cross-sectional |
| Al-Daghri et al., 2015 | 164 adults from Saudi Arabia | 83 patients with T2DM | 81 healthy controls | ↑ Serum irisin in T2DM than in controls. | Positive association between habitual physical activity and circulating irisin in healthy controls only; lack of association in T2DM patients suggests possible irisin dysregulation in metabolic dysfunction | Cross-sectional |
| García-Fontana et al., 2016 | 128 individuals from Spain | 73 patients with T2DM | 55 healthy controls | ↑ Serum irisin in T2DM than in controls. | Inverse relationship between irisin and myostatin levels; positive association between irisin and fasting plasma glucose and triglycerides in T2DM patients | Case-control |
| Wang et al., 2016 | 140 individuals from a university setting in China | 120 T2DM patients | 20 healthy controls with normal glucose tolerance | ↓ Serum irisin in T2DM patients than in healthy controls. | Positive correlation between irisin levels and insulin sensitivity; no significant association between irisin and pancreatic β-cell | Cross-sectional |
| Hwang et al., 2016 | 424 participants from South Korea | 137 with type 2 diabetes, 152 with prediabetes | 135 with normal glucose tolerance | ↓ Serum irisin levels were in T2DM and prediabetes groups compared to those with normal glucose tolerance. | Positive correlation between irisin levels and healthy metabolic parameters, such as reduced obesity, lower blood pressure, and improved glucose tolerance; inverse association with prediabetes/type 2 diabetes risk | Cross-sectional |
| Assyov et al., 2016 | 160 individuals from a university setting in Bulgaria | 60 with prediabetes, 50 with T2DM | 50 with normal glucose tolerance | ↓ Serum irisin levels progressively decreased from normal glucose tolerance to prediabetes, with the lowest levels in T2DM patients. | Negative correlations between irisin and fasting plasma glucose, positive correlation with IMC | Cross-sectional |
| Rana et al., 2017 | 79 T2DM patients from the UK | 79 T2DM patients with fasting plasma measurements | Reference values from healthy volunteers | ↑ Plasma irisin levels in T2DM patients compared to healthy controls. | Positive correlation between elevated plasma irisin levels and E-selectin, indicating potential endothelial dysfunction in T2DM patients | Cross-sectional |
| Li et al., 2017 | 462 adults from China | 362 patients with T2DM | 100 healthy controls | ↓ Serum irisin levels in T2DM patients compared to healthy controls. | Lower irisin levels were associated with increased skin autofluorescence values, indicating a higher accumulation of Advanced Glycation End-products (AGEs) in T2DM patients | Cross-sectional |
| El-Lebedy et al., 2018 | 205 subjects from Egypt | 135 T2DM patients | 70 healthy controls | ↓ Serum irisin in T2DM patients than healthy controls. | Negative correlation between irisin levels and HbA1c, HOMA-IR, and triglycerides in CVD patients; lower  hi irisin levels associated with 1.6 times increased risk of CVD in T2DM patients | Case-control |
| Elizondo-Montemayor et al., 2019 | 40 children and adolescents from Mexico | 20 patients with T2DM | 20 healthy controls | ↓ Plasma irisin in pediatric T2DM patients compared to healthy controls. | Positive correlation between irisin and HDL-c | Cross-sectional |
| AlKhairi et al., 2019 | 228 individuals from Kuwait | 104 T2DM patients | 124 non-diabetic individuals | ↑ Plasma irisin in T2DM than non-diabetic. | Positive correlation between irisin and Meteorin-like hormone; higher irisin levels in obese individuals compared to non-obese | Cross-sectional |
| Mostafa et al., 2021 | 120 individuals from a university setting in Egypt | 60 patients with newly diagnosed T2DM | 60 healthy controls | ↓ Serum irisin levels in T2DM patients compared to healthy controls | Negative correlation between irisin levels and fasting blood glucose, fasting plasma insulin, insulin resistance (HOMA-IR), and glycated hemoglobin | Cross-sectional |
| He et al., 2021 | 322 adults with newly diagnosed prediabetes from China | 161 participants who developed diabetes | 161 individuals with prediabetes | ↑ Serum irisin levels at baseline in prediabetic individuals who progressed to T2DM compared to those who did not. | A greater decrease in irisin levels during follow-up was correlated with an increased risk of diabetes | Nested case-control |
| Yildiz Kopuz et al., 2022 | 76 individuals from Turkey | 38 newly diagnosed T2DM patients | 38 healthy controls | ↑ Serum irisin in newly diagnosed T2DM patients compared to healthy controls. | Positive association between irisin levels and fruit intake in T2DM patients, no association with overall diet quality | Case-control |
| Mathia et al., 2023 | 30 participants from a university setting in Brazil | 16 T2DM patients | 14 healthy controls | → No significant difference in plasma irisin levels in T2DM patients and healthy controls. | Positive correlation between irisin gene expression and BMI in T2DM patients; negative correlation between plasma irisin and BMI | Case-control |
| Ahmed et al., 2023 | 90 participants from a university setting in Egypt | 65 patients with T2DM | 25 healthy controls | → No significant difference in serum irisin levels between T2DM patients and healthy controls | Significant inverse correlation between irisin levels and hs-CRP and LDL | Case-control |
| Type 2 Diabetes Only - Interventional | | | | | | |
| Liu et al., 2016 | 108 participants from China | 54 patients treated with exenatide for 12 weeks | 54 healthy controls with normal glucose tolerance | ↓ Serum irisin in T2DM at baseline; ↑ after exenatide. | The increase in irisin levels was significantly correlated with decreases in fasting blood glucose and HbA1c after exenatide treatment | Interventional study |
| Li et al., 2016 | 33 participants from China | 20 patients with T2DM treated with continuous subcutaneous insulin infusion for 1 week | 13 healthy controls | ↑ Serum irisin concentrations in T2DM patients after insulin infusion compared to pre-treatment. | The increase in irisin levels was associated with improvements in lipid profiles and glycemic control | Interventional study |
| Type 2 Diabetes and Associated Conditions | | | | | | |
| Type 2 Diabetes and Obesity / Metabolic syndrome | | | | | | |
| Shoukry et al., 2016 | 300 participants from Egypt | 150 T2DM patients and non-diabetic controls stratified into lean, overweight, and obese groups | 150 healthy controls, subdivided into lean, overweight and obese individuals. | ↓ Serum irisin in T2DM than in non-diabetic controls.  ↑ in obese non-diabetics than in lean controls. | Positive correlation between irisin and IMC. Negative correlation with insulin sensitivity. | Cross-sectional |
| Kamenov et al., 2017 | 128 men with metabolic syndrome from a university setting in Bulgaria | 90 men with T2DM | 38 men without T2DM | ↑ Serum irisin levels in men with hypogonadism and metabolic syndrome, especially in those with T2DM. | Significant negative correlation between irisin levels and serum testosterone; irisin was a predictor of hypogonadism | Nested case-control |
| Huerta-Delgado et al., 2020 | 56 children and adolescents from Mexico | 21 T2DM patients and 19 Metabolic Syndrome | 16 healthy controls | ↓ Serum irisin levels in both T2DM and Metabolic Syndrome patients compared to healthy controls | Negative correlation between irisin levels and BMI, waist circumference, and triglycerides; positive correlation with total cholesterol, HDL, and LDL | Cross-sectional |
| Cherian et al., 2021 | 228 individuals from Kuwait | 104 T2DM patients (66 obese, 38 non-obese) | 124 non-diabetic controls (51 obese, 73 non-obese) | ↑ Plasma irisin in T2DM and obese individuals compared to non-diabetic controls. | Irisin positively correlated with osteoactivin and osteoprotegerin. | Cross-sectional |
| Type 2 Diabetes and Diabetic retinopathy | | | | | | |
| Hu et al., 2016 | 178 T2DM patients from China | 35 patients with proliferative diabetic retinopathy, 22 with T2DM without retinopathy | 62 healthy controls | ↓ Serum irisin in T2DM with nephropathy, lowest in macroalbuminuria.  ↓ Serum and vitreous irisin in proliferative retinopathy | Negative correlation between serum irisin levels and indicators of renal function (creatinine, urea) and insulin resistance. | Cross-sectional |
| Wang et al., 2016 | 120 individuals from a university setting in China | 40 patients with nonproliferative diabetic retinopathy (NPDR), 60 T2DM patients without non-DR | 20 normal glucose tolerance controls | ↓ Serum irisin in T2DM than in normal glucose tolerance. → No difference between NPDR and non-DR groups. | Negative correlation between irisin and IL-17A levels; potential protective role of irisin against DR through anti-inflammatory effects | Cross-sectional |
| Tarboush et al., 2021 | 233 patients from a university setting in Jordan | 105 T2DM patients with diabetic retinopathy | 94 T2DM patients without diabetic retinopathy and 34 healthy controls | ↓ Plasma irisin in T2DM patients with diabetic retinopathy compared to those without retinopathy. | Negative correlation between irisin levels and HbA1c, triglycerides, and LDL; positive correlation with HDL | Case-control |
| Type 2 Diabetes and Associated Conditions (Nephropathy/renal complications) | | | | | | |
| Khidr et al., 2017 | 150 male adults from a university setting in Egypt | 60 T2DM patients with diabetic nephropathy, 40 T2DM patients with normoalbuminuria | 50 healthy controls | ↓ Serum irisin in T2DM patients, with a more pronounced decrease in those with diabetic nephropathy. | Significant correlations between irisin and metabolic parameters | Cross-sectional |
| Shelbaya et al., 2018 | 90 subjects from a university setting in Egypt | 30 patients with diabetic nephropathy, 30 patients with T2DM without complications | 30 healthy controls | ↓ Serum irisin in T2DM patients, with further reduction in those with diabetic nephropathy, compared to healthy controls. | Negative correlation between irisin levels and serum creatinine. Duration of diabetes was the strongest independent predictor of irisin levels | Case-control |
| Type 2 Diabetes and Cardiovascular diseases | | | | | | |
| Saadeldin et al., 2018 | 69 female subjects from Egypt | 39 T2DM patients with atherosclerosis, 22 T2DM patients without atherosclerosis | 8 healthy controls | ↓ Serum irisin in T2DM patients with atherosclerosis compared to those without and healthy controls. | Positive correlation between irisin and fasting blood glucose in T2DM patients with atherosclerosis | Case-control |
| Li et al., 2019 | 514 participants from China | 424 T2DM patients subdivided into 4 groups based on hypertension grades | 90 healthy controls | ↓ Serum irisin in T2DM patients, with further reduction in those with hypertension, compared to healthy controls. | Irisin levels were negatively correlated with blood pressure, fasting blood glucose, and leptin levels | Cross-sectional |
| Ali et al., 2020 | 138 subjects from Saudi Arabia | 46 T2DM patients with macrovascular complications | 46 T2DM patients without macrovascular complications, 46 healthy controls | ↓ Serum irisin in T2DM patients with macrovascular complications compared to those without and healthy controls. | Negative correlations between irisin and fasting blood glucose, insulin, HOMA-IR, HbA1c, and LDL | Case-control |
| Shanaki et al., 2017 | 162 subjects from Iran | 41 with nonalcoholic fatty liver disease, 41 with type 2 diabetes, and 40 with both nonalcoholic fatty liver disease and type 2 diabetes | 40 healthy controls | ↓ Serum irisin levels in all patient groups (NAFLD, T2DM, and both) compared to healthy controls. | Negative correlation of irisin with IMC, positive correlation with adiponectin levels | Cross-sectional |

**SM7. Summary of the included studies analyzing irisin levels in individuals with cardiovascular diseases.**

| Author/Year | Population/ Study location | Intervention/ Exposure | | Control | Outcome | | Conclusions | Study design | | |
| --- | --- | --- | --- | --- | --- | --- | --- | --- | --- | --- |
| Hypertension | | | | | | | | | | |
| Celik et al., 2015 | 115 patients from Turkey | 85 newly diagnosed, untreated hypertensive patients | | 30 age- and BMI-matched healthy controls without hypertension | ↓ Serum irisin in hypertensive patients vs. controls; ↑ after treatment with valsartan or amlodipine; → no difference between drugs. | | Both antihypertensive treatments elevated irisin, suggesting a potential role in energy homeostasis regulation in hypertension management. | Randomized controlled trial | | |
| Wang et al., 2023 | 300 patients from a university setting in China | 194 hypertensive patients | | 106 normotensive patients | ↓ Serum irisin in hypertensive dialysis patients compared to normotensive. | | Serum irisin levels showed a negative correlation with systolic blood pressure and pulse pressure; irisin, lean tissue mass, and serum calcium were independent predictors of systolic blood pressure | Cross-sectional | | |
| Peripheral Arterial Disease | | | | | | | | | | |
| Cao et al., 2025 | 202 individuals from China | | 112 patients with peripheral arterial disease | 90 healthy individuals | ↓ Serum irisin levels in PAD patients compared to healthy controls. | | Irisin levels were reduced in PAD. The study suggests irisin as a potential biomarker and predictor for PAD development. Associated with higher Rutherford classification and smoking. associated with HDL levels. | Case-control | | |
| Carotid Stenosis/Disease and Subclinical Atherosclerosis | | | | | | | | | | |
| Deng et al., 2016 | 564 participants from China | | 350 patients with coronary artery disease (CAD) confirmed by angiography | 214 healthy subjects | ↓ Serum irisin levels in CAD patients compared to healthy controls. | | Decreased serum irisin may be associated with CAD presence and severity | Cross-sectional | | |
| Remuzgo-Martínez et al., 2022 | 725 patients from Spain | | Patients with carotid plaques and/or atherogenic index ≥4 | Patients without carotid plaques and atherogenic index <4 | ↓ Serum irisin in patients with carotid plaques and worse lipid profile. | | Irisin levels were reduced in patients with worse cardiovascular risk and subclinical atherosclerosis, indicating a potential protective and biomarker role in axial spondyloarthritis. | Cohort study | | |
| Carmona-Maurici et al., 2023 | 61 participants from a university setting in Spain | | 30 participants with atheromatous plaques | 31 participants without atheromatous plaques | ↓ Serum irisin in participants with atheromatous plaques than in those without. | | Irisin levels were negatively correlated with carotid intima-media thickness and other atherogenesis-related parameters. | Prospective | | |
| Fu & Xing, 2024 | 236 individuals from China | | 156 patients with CAS, divided by BMI | 80 healthy adults with normal BMI | ↓ Serum irisin levels in CAS patients compared to healthy controls. | | Irisin levels were reduced in patients with CAS, especially those with overweight and obesity. Irisin was associated with lipid metabolism and severity of atherosclerosis, suggesting potential as a biomarker. | Cross-sectional | | |
| Major Cardiovascular Events (Acute Myocardial Infarction/ Heart Failure/Others) | | | | | | | | | | |
| Emanuele et al., 2014 | 437 participants from Italy and Spain | | 79 healthy centenarians, 178 young patients with early myocardial infarction | 180 age- and sex-matched healthy young controls | ↑ Serum irisin in centenarians; ↓ in young MI patients; → intermediate levels in young healthy controls | | Elevated irisin in centenarians may indicate its role in vascular protection and lifespan extension, whereas lower levels in MI patients could relate to cardiovascular risk | Cross-sectional | | |
| Aydin et al., 2014 | 25 subjects from a university setting in Turkey | | 11 patients with acute myocardial infarction (AMI) | 14 healthy controls | ↓ Irisin in both saliva and serum in AMI patients, especially within the first 48h. | | Irisin in saliva and serum may provide additional diagnostic information for AMI | Case-control | | |
| Anastasilakis et al., 2017 | 114 patients from Greece | | 31 with myocardial infarction (MI) and 40 with stable coronary artery disease (CAD) | 43 healthy controls without stenosis on angiography. | ↓ Serum irisin levels in MI and CAD patients compared to healthy controls. | | Decreased irisin in MI and CAD suggests reduced production due to limited blood supply | Non-randomized, interventional study | | |
| Abd El-Mottaleb et al., 2020 | 86 participants from a university setting in Egypt | | 33 with MI, 33 with MI and heart failure | 20 controls | ↓ Serum irisin in MI and HF groups compared to controls. | | Low irisin levels may serve as a biomarker for MI severity and correlate with inflammation and dyslipidemia, supporting its potential role in early cardiovascular risk assessment. | Case-control | | |
| Önalan Etem et al., 2021 | 450 patients from a university setting in Turkey | | 225 patients with acute myocardial infarction (AMI) | 225 healthy controls | ↓ Serum irisin in AMI patients compared to controls, especially within 24h post-AMI. | | Irisin levels decreased in AMI. FNDC5 gene variants were mostly non-significant for irisin levels in AMI context. | Case-control | | |
| Berezin et al., 2022 | 208 patients from Ukraine | 153 patients with heart failure | | 30 patients with T2DM without heart failure and 25 healthy volunteers | | ↓ Serum irisin in HF patients compared to T2DM without HF. | Irisin levels improved the predictive ability for heart failure with preserved ejection fraction, but not for heart failure with mildly reduced ejection fraction or heart failure with reduced ejection fraction | Prospective cohort | | |
| Huerta-Delgado et al., 2022 | 64 patients from Mexico | 32 patients with chronic heart failure under standard treatment (CHF) | | 32 healthy controls | ↓ Serum irisin in CHF patients compared to controls. | | Negative correlations were observed between irisin and brain natriuretic peptide, insulin levels, and insulin resistance index; no significant correlations were found with inflammatory cytokines | Cross-sectional | | |
| Tu et al., 2018 | 1,325 patients from China | 1,205 Chinese patients with first-ever acute ischemic stroke (AIS) | | 120 healthy controls without psychiatric or neurological disorders | ↓ Serum irisin in AIS patients with post-stroke depression | | Reduced irisin levels may serve as an independent predictor of PSD risk | Cohort study | | |
| Dong et al., 2021 | 152 patients from a university setting in China | Group with cardiovascular/cerebrovascular diseases: 47 patients | | Group without cardiovascular/cerebrovascular diseases: 105 patients | ↓ Serum irisin associated with cardiovascular and cerebrovascular mortality. | | Cox regression identified lower irisin levels as an independent predictor for cardiovascular and cerebrovascular mortality, but not for all-cause mortality | Retrospective cohort study | | |
| Chai et al., 2023 | 207 patients from China | 86 patients who developed major adverse cardiovascular events within 1 year after intervention | | 121 patients without major adverse cardiovascular events | ↓ Serum irisin levels in MACE group compared to non-MACE. | | Irisin levels at admission were negatively correlated with AMI severity and predicted MACE with an AUC of 0.75 | Retrospective cohort study | | |
| Other Diseases (Behçet’s Disease/ Acromegaly) | | | | | | | | | | |
| Icli et al., 2016 | 98 participants from a university setting in Turkey | | 48 patients with Behçet's disease (BD) | 50 healthy controls with no regular medications or known diseases. | ↓ Serum irisin levels in Behçet’s disease patients compared to healthy controls. | | Low serum irisin levels may independently predict subclinical atherosclerosis in BD | | Prospective study | |
| Ismail et al., 2023 | 50 patients from a university setting in Egypt | | 50 patients with Behçet’s disease | 50 healthy controls | ↓ Serum irisin in Behçet’s disease patients, particularly those with subclinical atherosclerosis. | | Lower irisin levels in Behçet’s disease patients, especially those with subclinical atherosclerosis, suggest its potential role as a protective biomarker against vascular dysfunction. | | Case-control | |
| Calan & Demirpence, 2019 | 110 participants from Turkey | | 40 with active acromegaly, 30 with controlled acromegaly | 40 healthy controls | ↑ Serum irisin in active and controlled acromegaly. | | Elevated irisin levels in patients with acromegaly are associated with cardiovascular risk parameters | | | Cross-  sectional |

**SM8.** Funnel plots of included studies within each condition. Each study is represented by a bar proportionate to the study’s sample size. Green bars indicate lower serum/plasma irisin levels in disease; red indicates higher irisin levels in disease; and yellow indicates no statistically significant difference. Interventional studies in diabetes were not included due to specific drug-related comparisons.

**Disclosure**

This manuscript did not use artificial intelligence in any capacity.

**Conflicts of Interest**

The authors declare no conflicts of interest.

**REFERENCES**

Abd El-Mottaleb NA, Galal HM, El Maghraby KM, Gadallah AI (2019) Serum irisin level in myocardial infarction patients with or without heart failure. Can J Physiol Pharmacol 97:932–938. https://doi.org/10.1139/cjpp-2018-0736

Ahmed TM, Nassar M, Mohamed HAA, et al (2023) Evaluation of serum levels of Irisin as a marker of endothelial dysfunction in patients with type 2 diabetes mellitus. Endocrinol Diabetes Metab 6:e0403. https://doi.org/10.1002/edm2.403

Al‐Daghri NM, Alokail MS, Rahman S, et al (2015) Habitual physical activity is associated with circulating irisin in healthy controls but not in subjects with diabetes mellitus type 2. Eur J Clin Invest 45:775–781. https://doi.org/10.1111/eci.12468

Ali EY, Hegazy GA, Hashem EM (2020) Evaluation of irisin, retinol-binding protein 4, and leptin serum levels as biomarkers of macrovascular complications involvement in Saudi type 2 diabetes mellitus. A case-control study. Saudi Med J 41:1369–1374. https://doi.org/10.15537/smj.2020.12.25461

Alis R, Sanchis-Gomar F, Pareja-Galeano H, et al (2014) Association between irisin and homocysteine in euglycemic and diabetic subjects. Clin Biochem 47:333–335. https://doi.org/10.1016/j.clinbiochem.2014.08.017

AlKhairi I, Cherian P, Abu-Farha M, et al (2019) Increased Expression of Meteorin-Like Hormone in Type 2 Diabetes and Obesity and Its Association with Irisin. Cells 8:1283. https://doi.org/10.3390/cells8101283

Anastasilakis AD, Polyzos SA, Saridakis ZG, et al (2014) Circulating Irisin in Healthy, Young Individuals: Day-Night Rhythm, Effects of Food Intake and Exercise, and Associations With Gender, Physical Activity, Diet, and Body Composition. J Clin Endocrinol Metab 99:3247–3255. https://doi.org/10.1210/jc.2014-1367

Assyov Y, Gateva A, Tsakova A, Kamenov Z (2016) Irisin in the Glucose Continuum. Experimental and Clinical Endocrinology & Diabetes 124:22–27. https://doi.org/10.1055/s-0035-1564130

Aydin S, Aydin S, Kobat MA, et al (2014) Decreased saliva/serum irisin concentrations in the acute myocardial infarction promising for being a new candidate biomarker for diagnosis of this pathology. Peptides (NY) 56:141–145. https://doi.org/10.1016/j.peptides.2014.04.002

Berezin AA, Lichtenauer M, Boxhammer E, et al (2022) Discriminative Value of Serum Irisin in Prediction of Heart Failure with Different Phenotypes among Patients with Type 2 Diabetes Mellitus. Cells 11:2794. https://doi.org/10.3390/cells11182794

Calan M, Demirpence M (2019) Increased circulating levels of irisin are associated with cardiovascular risk factors in subjects with acromegaly. Hormones 18:435–442. https://doi.org/10.1007/s42000-019-00151-3

Cao Y, Wei T, Li Y, Duan H (2025) Serum Irisin as a Predictor for Peripheral Arterial Disease: Insights from a Clinical Study. Ann Vasc Surg 111:70–82. https://doi.org/10.1016/j.avsg.2024.10.017

Carmona-Maurici J, Rosa A, Azcona-Granada N, et al (2023) Irisin as a Novel Biomarker of Subclinical Atherosclerosis in Severe Obesity. Int J Mol Sci 24:8171. https://doi.org/10.3390/ijms24098171

Celik H, Akkaya N, Erdamar H, et al (2015) The Effects of Valsartan and Amlodipine on the Levels of Irisin, Adropin, and Perilipin. Clin Lab 61:1889–1895. https://doi.org/10.7754/Clin.Lab.2015.150420

Chai Q, Zhang W, Gao L, et al (2023) Serum irisin correlates to the severity of acute myocardial infarction and predicts the postoperative major adverse cardiovascular events. Biomolecules and Biomedicine 23:911–919. https://doi.org/10.17305/bb.2023.8888

Cherian P, Al-Khairi I, Jamal M, et al (2021) Association Between Factors Involved in Bone Remodeling (Osteoactivin and OPG) With Plasma Levels of Irisin and Meteorin-Like Protein in People With T2D and Obesity. Front Endocrinol (Lausanne) 12:752892. https://doi.org/10.3389/fendo.2021.752892

Choi Y-K, Kim M-K, Bae KH, et al (2013) Serum irisin levels in new-onset type 2 diabetes. Diabetes Res Clin Pract 100:96–101. https://doi.org/10.1016/j.diabres.2013.01.007

Cuttitta G, Ferraro M, Cibella F, et al (2023) Relationship among Body Composition, Adipocytokines, and Irisin on Exercise Capacity and Quality of Life in COPD: A Pilot Study. Biomolecules 13:48. https://doi.org/10.3390/biom13010048

Deng W (2016) Association of Serum Irisin Concentrations with Presence and Severity of Coronary Artery Disease. Medical Science Monitor 22:4193–4197. https://doi.org/10.12659/MSM.897376

Dong X, Fu W, Deng Y, et al (2021) Lower serum irisin levels are associated with the increasing mortality of cardiovascular and cerebrovascular diseases in hemodialysis patients. Ann Palliat Med 10:6052–6061. https://doi.org/10.21037/apm-21-406

Duran ID, Gülçelik NE, Ünal M, et al (2015) Irisin levels in the progression of diabetes in sedentary women. Clin Biochem 48:1268–1272. https://doi.org/10.1016/j.clinbiochem.2015.07.098

El-Lebedy DH, Ibrahim AA, Ashmawy IO (2018) Novel adipokines vaspin and irisin as risk biomarkers for cardiovascular diseases in type 2 diabetes mellitus. Diabetes & Metabolic Syndrome: Clinical Research & Reviews 12:643–648. https://doi.org/10.1016/j.dsx.2018.04.025

Elizondo-Montemayor L, Gonzalez-Gil AM, Tamez-Rivera O, et al (2019) Association between Irisin, hs-CRP, and Metabolic Status in Children and Adolescents with Type 2 Diabetes Mellitus. Mediators Inflamm 2019:1–13. <https://doi.org/10.1155/2019/6737318>

Emanuele E, Minoretti P, Pareja-Galeano H, Sanchis-Gomar F, Garatachea N, Lucia A (2014) Serum irisin levels, precocious myocardial infarction, and healthy exceptional longevity. Am J Med 127:888-890. doi:10.1016/j.amjmed.2014.04.025

Fu S, Xing G (2024) Changes in Serum Irisin Levels and Their Significance in Carotid Atherosclerosis Associated with Obesity. Alternative therapies in health and medicine 30:194–199.

García-Fontana B, Reyes-García R, Morales-Santana S, et al (2016) Relationship between myostatin and irisin in type 2 diabetes mellitus: a compensatory mechanism to an unfavourable metabolic state? Endocrine 52:54–62. https://doi.org/10.1007/s12020-015-0758-8

He X, Zhang Q, Peng N, et al (2021) Irisin plays an important role in the outcomes of newly diagnosed prediabetes in adults in Guiyang, China. J Diabetes Investig 12:747–755. https://doi.org/10.1111/jdi.13416

Hu W, Wang R, Li J, et al (2016) Association of irisin concentrations with the presence of diabetic nephropathy and retinopathy. Annals of Clinical Biochemistry: International Journal of Laboratory Medicine 53:67–74. https://doi.org/10.1177/0004563215582072

Huerta-Delgado AS, Roffe-Vazquez DN, Gonzalez-Gil AM, et al (2020) Serum Irisin Levels, Endothelial Dysfunction, and Inflammation in Pediatric Patients with Type 2 Diabetes Mellitus and Metabolic Syndrome. J Diabetes Res 2020:1–16. https://doi.org/10.1155/2020/1949415

Huerta-Delgado AS, Roffe-Vazquez DN, Luna-Ceron E, et al (2022) Association of irisin levels with cardiac magnetic resonance, inflammatory, and biochemical parameters in patients with chronic heart failure versus controls. Magn Reson Imaging 93:62–72. https://doi.org/10.1016/j.mri.2022.07.006

Hwang Y-C, Jeon WS, Park C-Y, Youn B-S (2016) The ratio of skeletal muscle mass to visceral fat area is a main determinant linking circulating irisin to metabolic phenotype. Cardiovasc Diabetol 15:9. https://doi.org/10.1186/s12933-015-0319-8

Icli A, Cure E, Cumhur Cure M, et al (2016) Novel myokine: irisin may be an independent predictor for subclinic atherosclerosis in Behçet's disease. J Investig Med 64:875-881. doi:10.1136/jim-2015-000044

Ijiri N, Kanazawa H, Asai K, et al (2015) Irisin, a newly discovered myokine, is a novel biomarker associated with physical activity in patients with chronic obstructive pulmonary disease. Respirology 20:612–617. https://doi.org/10.1111/resp.12513

Ismail MA, Mounir O, Sedky A, et al (2023) Exists a role for serum irisin in Egyptian Behcet’s patients with subclinical atherosclerosis? Clin Rheumatol 42:179–186. https://doi.org/10.1007/s10067-022-06368-9

Kamenov Z, Assyov Y, Angelova P, et al (2017) Irisin and Testosterone in Men with Metabolic Syndrome. Hormone and Metabolic Research 49:755–759. https://doi.org/10.1055/s-0043-115227

Kaya H, Boyaci H, Argun Baris S, et al (2024) The predictive effects of adiponectin and irisin hormones on diagnosis and clinical involvement of Sarcoidosis. BMC Pulm Med 24:623. https://doi.org/10.1186/s12890-024-03412-z

Khidr EG, Ali SS, Elshafey MM, Fawzy OA (2017) Association of irisin and FNDC5 rs16835198 G &gt; T gene polymorphism with type 2 diabetes mellitus and diabetic nephropathy. An Egyptian pilot study. Gene 626:26–31. https://doi.org/10.1016/j.gene.2017.05.010

Li B, Yao Q, Guo S, et al (2019) Type 2 diabetes with hypertensive patients results in changes to features of adipocytokines: Leptin, Irisin, LGR4, and Sfrp5. Clin Exp Hypertens 41:645–650. https://doi.org/10.1080/10641963.2018.1529779

Li L, Rampersad S, Wang X, et al (2016) Serum irisin concentrations were increased after transient continuous subcutaneous insulin infusion in type 2 diabetes mellitus patients. Diabetes Res Clin Pract 113:44–47. https://doi.org/10.1016/j.diabres.2016.01.030

Li Z, Wang G, Zhu Y, et al (2017) The relationship between circulating irisin levels and tissues AGE accumulation in type 2 diabetes patients. Biosci Rep 37:BSR20170213. https://doi.org/10.1042/BSR20170213

Liu J, Hu Y, Zhang H, et al (2016) Exenatide treatment increases serum irisin levels in patients with obesity and newly diagnosed type 2 diabetes. J Diabetes Complications 30:1555–1559. https://doi.org/10.1016/j.jdiacomp.2016.07.020

Mathia VL, Mendonça MIS, Simões DP, et al (2023) Relationship of irisin expression with metabolic alterations and cardiovascular risk in type 2 diabetes mellitus: a preliminary study. Rev Assoc Med Bras 69:e0812. https://doi.org/10.1590/1806-9282.20230812

Mostafa TM, El-Gharbawy NM, Werida RH (2021) Circulating IRAPe, Irisin, and IL-34 in Relation to Insulin Resistance in Patients With Type 2 Diabetes. Clin Ther 43:e230–e240. https://doi.org/10.1016/j.clinthera.2021.05.003

Önalan Etem E, Dis O, Tektemur A, et al (2021) Common single nucleotide polymorphisms in the FNDC5 gene and serum irisin levels in acute myocardial infarction. The Anatolian Journal of Cardiology 25:528–535. https://doi.org/10.5152/AnatolJCardiol.2021.36214

Rana KS, Pararasa C, Afzal I, et al (2017) Plasma irisin is elevated in type 2 diabetes and is associated with increased E-selectin levels. Cardiovasc Diabetol 16:147. https://doi.org/10.1186/s12933-017-0627-2

Remuzgo-Martínez S, Rueda-Gotor J, Pulito-Cueto V, et al (2022) Irisin as a Novel Biomarker of Subclinical Atherosclerosis, Cardiovascular Risk and Severe Disease in Axial Spondyloarthritis. Front Immunol 13:894171. https://doi.org/10.3389/fimmu.2022.894171

Saadeldin MK, Elshaer SS, Emara IA, et al (2018) Serum sclerostin and irisin as predictive markers for atherosclerosis in Egyptian type II diabetic female patients: A case control study. PLoS One 13:e0206761. https://doi.org/10.1371/journal.pone.0206761

Shanaki M, Moradi N, Emamgholipour S, et al (2017) Lower circulating irisin is associated with nonalcoholic fatty liver disease and type 2 diabetes. Diabetes & Metabolic Syndrome: Clinical Research & Reviews 11:S467–S472. https://doi.org/10.1016/j.dsx.2017.03.037

Shelbaya S, Abu Shady MM, Nasr MS, et al (2018) Study of Irisin Hormone Level in Type 2 Diabetic Patients and Patients with Diabetic Nephropathy. Curr Diabetes Rev 14:481–486. https://doi.org/10.2174/1573399813666170829163442

Shiotani T, Sugimoto S, Yamamoto H, et al (2022) Emphysematous changes and lower levels of plasma irisin are associated with bronchiolitis obliterans syndrome after bilateral living-donor lobar lung transplantation. Surg Today 52:294–305. https://doi.org/10.1007/s00595-021-02339-w

Shoukry A, Shalaby SM, El‐Arabi Bdeer S, et al (2016) Circulating serum irisin levels in obesity and type 2 diabetes mellitus. IUBMB Life 68:544–556. https://doi.org/10.1002/iub.1511

Tarboush NA, Abu-Yaghi NE, Al Ejeilat LH, et al (2021) Association of Irisin Circulating Level with Diabetic Retinopathy: A Case-Control Study. Experimental and Clinical Endocrinology & Diabetes 129:36–42. https://doi.org/10.1055/a-0723-3749

Tu W-J, Qiu H-C, Cao J-L, et al (2018) Decreased Concentration of Irisin Is Associated with Poor Functional Outcome in Ischemic Stroke. Neurotherapeutics 15:1158–1167. https://doi.org/10.1007/s13311-018-0651-2

Wang C, Wang L, Liu J, et al (2016) Irisin modulates the association of interleukin-17A with the presence of non-proliferative diabetic retinopathy in patients with type 2 diabetes. Endocrine 53:459–464. https://doi.org/10.1007/s12020-016-0905-x

[Wang H, Zhang X, Chen W, et al (2015) Relationship between serum irisin levels and urinary albumin excretion in patients with type 2 diabetes. J Diabetes Complications 29:384–389. https://doi.org/10.1016/j.jdiacomp.2015.01.001](https://doi.org/10.1016/j.jdiacomp.2015.01.001)

Wang L, Song J, Wang C, et al (2016) Circulating Levels of Betatrophin and Irisin Are Not Associated with Pancreatic β -Cell Function in Previously Diagnosed Type 2 Diabetes Mellitus Patients. J Diabetes Res 2016:1–8. https://doi.org/10.1155/2016/2616539

Wang P-X, Fan Z-J, Wu L-Y, et al (2023) Serum irisin levels are negatively associated with blood pressure in dialysis patients. Hypertension Research 46:2738–2745. https://doi.org/10.1038/s41440-023-01449-x

Xiang L, Xiang G, Yue L, et al (2014) Circulating irisin levels are positively associated with endothelium-dependent vasodilation in newly diagnosed type 2 diabetic patients without clinical angiopathy. Atherosclerosis 235:328–333. https://doi.org/10.1016/j.atherosclerosis.2014.04.036

Yildiz Kopuz TN, Dagdeviren M, Fisunoglu M (2022) Serum irisin levels in newly diagnosed type-II diabetic patients: No association with the overall diet quality but strong association with fruit intake. Clin Nutr ESPEN 49:357–364. https://doi.org/10.1016/j.clnesp.2022.03.022
